# Effect of Intrathecal Morphine on Postdural Puncture Headache in Obstetric Anaesthesia

**DOI:** 10.4274/TJAR.2023.221140

**Published:** 2023-08-18

**Authors:** Meryem Onay, Sema Şanal Baş, Arda Işıker, Ümit Akkemik, Ayten Bilir

**Affiliations:** 1 Department of Anaesthesiology and Reanimation, Eskişehir Osmangazi University Faculty of Medicine, Eskişehir, Turkey; 2 Clinic of Anaesthesiology and Reanimation, Kırklareli Training and Research Hospital, Kırklareli, Turkey; 3 Clinic of Algology, Eskişehir City Hospital, Eskişehir, Turkey

**Keywords:** Intrathecal morphine, obstetric anaesthesia, postdural puncture headache, postoperative analgesia, spinal anaesthesia

## Abstract

**Objective:**

Intrathecal morphine is used as an effective component of multimodal analgesia in postoperative analgesia in cesarean section patients. We aimed to analyze the relationship between intrathecal morphine administration and postdural puncture headache (PDPH), pain score and analgesia consumption in the postoperative period, and maternal fetal effects.

**Methods:**

One hundred four pregnant women aged ≥18 years (American Society of Anesthesiology physical status I or II, >36 weeks gestation) who were scheduled for elective cesarean section under spinal anaesthesia were included in this study. Spinal anesthesia consisted of bupivacaine with or without morphine (Group M: 10 mg heavy marcaine + 25 mcg fentanyl + 100 mcg morphine; Group F: 10 mg heavy marcaine + 25 mcg fentanyl). The effect of intrathecal morphine on PDPH, postoperative pain score, analgesia consumption, and maternal and fetal effects were recorded for 5 days.

**Results:**

PDPH developed in a total of 33 patients (Group M: 18 and Group F: 15, *P*=0.274). When we evaluated PDPH with the VAS, there was no significant difference between the groups. The postoperative visual analogue scale (VAS) was lower in the morphine group, and no statistically significant difference was found in the VAS 1^st^ hr and VAS 2^nd^ hr, whereas the VAS 6^th^ hr and VAS 24^th^ hr were found to be statistically significant. There was no difference in terms of PDPH, the first analgesic requirement and postoperative nausea-vomiting, but meperidine consumption was lower in the morphine group.

**Conclusion:**

Low-dose intrathecal morphine did not affect the incidence of PDPH. It is an effective method that can be used in cesarean section patients without increasing the maternal and fetal side effects from postoperative analgesia.

**Main Points**

• The administration of neuraxial morphine in postoperative analgesia is considered the gold standard and provides superior analgesia compared to systemic practice.

• Epidemiologically, postdural puncture headache (PDPH) is the most common complication and is observed in approximately 10-30% of patients with lumbar puncture, while the risk increases in young and obstetric patients.

• Low-dose intrathecal morphine is an efficient and effective component of multimodal analgesia without increasing maternal and fetal side effects in cesarean section patients.

• Low-dose 0.1 mg morphine had no protective efficacy in reducing PDPH incidence and pain severity.

## Introduction

The use of neuraxial techniques has increased because of their safer and postoperative analgesia contribution compared with general anaesthesia at childbirth.^1^ Morphine, which is applied with neuraxial blocks (spinal, epidural anaesthesia) during cesarean section, is an effective and easily applicable part of postoperative multimodal analgesia. Considering the risk factors for the patient, it is recommended that a low dose of morphine can be used safely in postoperative analgesia during cesarean section when the respiratory monitoring frequency and duration are monitored.^2^ Intrathecal morphine exerts its analgesic efficacy through opioid receptors, which are also found in the substantia gelatinosa in the dorsal horn of the spinal cord. The respiratory depression effect of neuroaxial morphine administration is biphasic. In epidurally administered morphine, systemic absorption occurs in the early period (30-90 min), and in epidurally or intrathecally administered morphine, late period effects (6-18 hours) occur with rostral spread into the cerebrospinal fluid and with slow penetration into the brain stem. Its low fat resolution explains why its effect starts slowly and lasts for a long time.^3^

During cesarean section, spinal anaesthesia is the first choice owing to advantages such as simplicity of the technique, rapid application, rapid action start, increased block density, and low risk of systemic toxicity compared to other techniques.^4,5^ Epidemiologically, PDPH is the most common complication and is observed in approximately 10-30% of patients with lumbar puncture, while the risk increases in young and obstetric patients.^1^ The mother’s inability to meet the needs of her baby is a cause of morbidity, which increases the length of hospital stay and health care costs.^6^ PDPH is usually benign and not long term but is sometimes associated with chronic headaches and back pain.^7^ Recommendations on risk factors and treatment for PDPH have been identified, and the effect of neuraxial morphine on prophylaxis has been discussed.

Our primary objective in the present study was to assess the relationship between low-dose intrathecal morphine administration and postdural puncture headache (PDPH). Our secondary aim was to analyze the need for the first analgesia, meperidine consumption, maternal visual analogue scale (VAS) and side effects (nausea, vomiting, itching, and respiratory depression) in the postoperative period.

## Methods

Because of the high incidence of PDPH in the pregnant population, pregnant women who have neuraxial blocks in our clinic are closely monitored, and regular records are kept. This study was planned retrospectively. The data of 128 patients were examined after obtaining approval from the Eskişehir Osmangazi University Non-Invasive Clinical Research Ethics Committee (approval no: 28, date: 12.01.2021). Twenty-four patients were excluded from the study due to missing documents or different doses of morphine administered. Pregnant women aged ≥18 years with a physical classification of American Society of Anesthesiology (ASA) I-II who underwent spinal anaesthesia for cesarean section under elective conditions were included in the study. A fentanyl group (Group F: 10 mg heavy marcaine + 25 mcg fentanyl) and a morphine group (Group M: 10 mg heavy marcaine + 25 mcg fentanyl + 0.1 mg morphine) were included in the study and had standardized doses. The patients’ age, body mass index (BMI), gestational week, migraine or other PDPH history, intrathecal opioid (fentanyl and morphine), level of spinal anaesthesia, number of trials, intraoperative hemodynamic response, perioperative complications, postoperative VAS, meperidine consumption and first analgesic requirement, pH of infant blood gas, Apgar score of the baby in the 1^st^ min and 5^th^ min, and PDPH presence and severity were checked from the records. PDPH is defined as bilateral and non-throbbing positional headache, occurring within 5 days after dural puncture, intensifies within 15 minutes in a sitting or standing position, decreases within 15 minutes in a back position and is usually present in the fronto-occipital position. Neck stiffness can be accompanied by vestibular, visual, and auditory symptoms.^8,9^

In our clinic, headache severity is evaluated using the VAS (0 represents no pain and 10 represents the worst pain), and patients are evaluated in the preoperative period. Patients with VAS <4 are considered to have mild headache, and conservative methods, such as bed rest, analgesia, oral caffeine and hydration, are recommended. In the patients who do not respond to these treatments, those who have VAS >4 and those who have challenges in infant care are referred to our algology clinic, and opioids, epidural blood patches or sphenopalatine ganglion blocks may be added to the standard pain control regimen. Clinically stable patients are discharged on the 2^nd^ day following the operation. Although the frequency of the patients who have follow-up evaluations is increased in the first 24 hours, the patient’s 5-day follow-up is completed with a phone call.

### Statistical Analysis

Assuming that a relative decrease of at least 50% would be necessary to support the clinical usefulness of this treatment, we determined that a sample size of 64 patients in each group would be required to achieve an 80% power to detect this difference between intrathecal morphine and fentanyl administration. Group sample sizes of 64 were required to achieve an 80% power in detecting a difference between the group proportions of 0.25. The proportion in the treatment group is assumed to be 0.5 under the null hypothesis and 0.25 under the alternative hypothesis. The proportion in the control group is 0.5. A superiority trial test was performed using the two-sided Fisher’s exact test. The significance level of the test was targeted at 0.05. The sample size was calculated using PASS 11, version 11.0.7, release date June 28, 2011 (NCSS Inc., USA).

Continuous data are given as the average ± standard deviation. Categorical data are given as percentages (%).

The Shapiro-Wilk test was used to investigate the compliance of the data with a normal distribution. In the comparison of the normally distributed variables, an Independent Samples t-test analysis was used to compare the variables between the two groups. The Mann-Whitney U test was used for the comparison of the variables that did not conform to the normal distribution to determine the differences between the two groups. Pearson’s chi-squared and Pearson’s exact chi-squared analyses were used in the analysis of the created cross tables. Logistic regression analysis was used to determine risk factors. In the implementation of the analyses, the IBM SPSS Statistics 21.0 (IBM Corp. Released 2011. IBM SPSS Statistics for Windows, Version 20.0. Armonk, NY: IBM Corp.) program was used. A value of *P* < 0.05 was accepted as the criterion for statistical significance.

## Results

**Figure 1:**
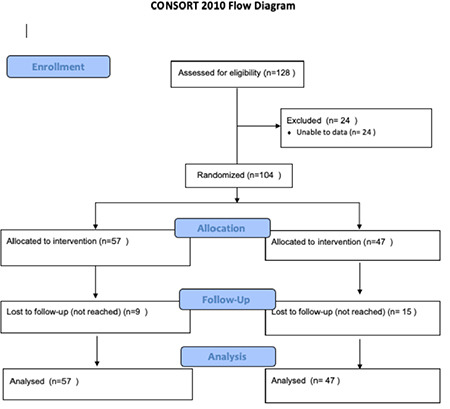
Consort flow diagram of patient flow in the study.

The study included 104 elective cesarean section patients with ASA I-II. The data of a total of 128 patients were analyzed. Since data from 24 patients were not available, these patients were excluded from the study (Figure 1). The patients were divided based on the doses given and into Group M (Group M: 10 mg heavy marcaine + 25 mcg fentanyl + 0.1 mg morphine) (n = 47) and Group F (Group F: 10 mg heavy marcaine + 25 mcg fentanyl) (n = 57) using the above standard doses. Demographic data (age, BMI, and gestational week), migraine and PDPH history are included in Table 1.

Spinal anaesthesia was administered in the L3-4 or L4-5 vertebral space. The number of attempts and the levels applied were similar between the groups (*P* > 0.05). There was no statistically significant difference between intraoperative hypotension (*P*=0.94), itching (*P*=0.89), nausea and vomiting (*P*=0.53), need for vasoconstrictor (*P*=0.87), and given fluid volumes (*P*=0.35).

There was no statistically significant difference in the newborn’s blood gas regarding pH (*P*=0.54) and Apgar score at the 1^st^ minute (*P*=0.93) and at the 5^th^ minute (*P*=0.79).

**Figure 2:**
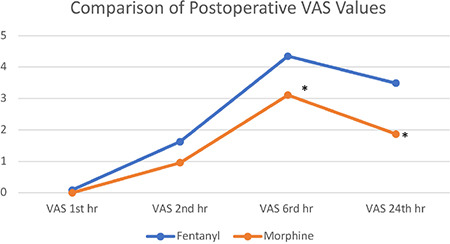
Comparison of postoperative VAS values. VAS, visual analogue scale.

The sensory block, Ramsay sedation scale, and Bromage scale checked postoperatively were similar in the 0^th^, 1^st^, and 6^th^ hours. The postoperative VAS scores were lower in the morphine group, and VAS in the 1^st^ hour (*P*=0.197), VAS in the 2^nd^ hour (*P*=0.23), VAS in the 6^th^ hour (*P*=0.01), and VAS in the 24^th^ hour (*P* < 0.001) were statistically significant (Figure 2).

**Figure 3:**
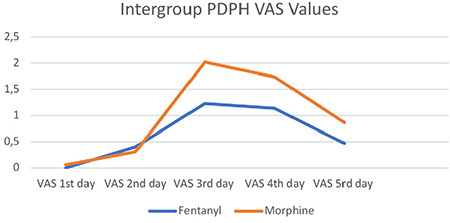
Intergroup PDPH VAS values. VAS, visual analogue scale; PDPH, postdural puncture headache.

There was no statistically significant difference between PDPH, the first analgesic requirement, postoperative nausea-vomiting, hospitalization duration, and patient satisfaction, but meperidine consumption was lower in the morphine group (Table 2). In-group PDPH was analyzed through VAS and was found to be similar between the groups (Figure 3) (Group M: 47 and Group F: 57, n = 104).

The incidence of PDPH was 38.3% (n = 18) in Group M and 26.3% (n = 15) in Group F. PDPH developed in a total of 33 patients, and given the risk factors for PDPH, it was observed that its incidence was increased in those with a history of PDPH (*P*=0.019) and in those with lower arterial blood pressure (*P*=0.017).

## Discussion

Neuroaxial morphine is considered the gold standard for postoperative analgesia in cesarean section. In the present study, 0.1 mg morphine was administered only intrathecally for postoperative analgesia, and the effect of low-dose morphine on PDPH incidence was not observed. The postoperative VAS scores were lower in the morphine group, and the VAS in the 6^th^ and 24^th^ hours was especially significant. Complications, such as perioperative nausea-vomiting and itching, were similar between the two groups.

In the present study, in the pregnant women, the spinal blocks were applied with routine 25 G Quincke needles. Conservative treatment was applied in PDPH treatment; only 2 patients from the morphine group were given opioids. PDPH occurs by the transition of BOS from the dural puncture region to the epidural and paravertebral area faster than BOS production. It is the most common complication, with an incidence of 36.5% after dural puncture. Female sex, low BMI, young age, large needle size, needle direction, number of lumbar punctures, and needle design (cutting-tip needles compared to pencil point needles) are risk factors. Twenty-five gauge cutting and noncutting-tipped spinal needles were used in the pregnant women. The incidence of PDPH was shown to be 36.7% in the group in which cutting-tipped needles were used and 6.7% in the group in which non-cutting needles were used.^10,11^ Conservative treatments such as analgesics, caffeine, hydration and bed rest can be used in the treatment of PDPH. However, in cases where conservative treatment is not effective in PDPH, the efficacy of prophylactic epidural blood patch, long-term intrathecal catheter placement, epidural or intrathecal morphine and epidural saline is discussed.^8,12^ In the morphine group, the incidence of PDPH was similar to that in the present study, and no reduction was observed.

Given an effective volume (10 cc saline) to the epidural space, slow systemic absorption of epidural morphine, and given it twice with an interval of 24-hour suggest that it may be effective in reducing the incidence of PDPH. The effect of epidural morphine on PDPH was evaluated in the Al-metwalli^13^ prospective randomized controlled study. The incidence of PDPH was 78-85% after accidental dural puncture with a 17 G epidural needle in obstetric patients. After accidental dural puncture in pregnant women with increased PDPH risk, 3 mg of morphine + 10 cc saline and 10 cc of saline were applied to the control group with a 24 hr interval and were placed at the same or different level of the epidural catheter. The PDPH incidence has been shown to be associated with reducing therapeutic blood patching and delaying the onset of PDPH symptoms in the epidural morphine group. Although the mechanism is unclear, the PDPH incidence decreased to 48% in the saline group and 12% in the morphine group.^13^

Diamorphine has a higher lipid resolution than morphine. It reaches neural tissues faster, and it is less commonly involved in central side effects such as respiratory depression due to the onset time and shortening of the half-life in the BOS.^14^ Spinal anaesthesia was performed with a 25 G Whitacre needle in the study, which analyzed 4559 cesarean patients. They were grouped into those who did not receive intrathecal opioids and those who received intrathecal 10-20 mcg fentanyl and 300 mcg diamorphine. The results show that reduced the incidence of PDPH was not associated with intrathecal fentanyl, but with diamorphine.^15^

In the retrospective study by Brinser et al.^16^, the patients were classified into Group M, who received epidural or intrathecal morphine during a cesarean section by placing a catheter after dural puncture, and Group C, who did not receive neuroaxial morphine. The act of childbirth (NVD or CS) was not standard. There was no decrease in PDPH risk, severity of headache or need for epidural blood patches in the patients with neuroaxial morphine.^16^ Although morphine doses are not standard in the present study, the applied areas (epidural and intrathecal) are different. As a result, there are studies that showed a reduced PDPH risk when an effective volume was given as an epidural.

Intrathecal route was preferred over the neuraxial pathway, and the morphine doses were kept constant. Although analgesic efficacy of intrathecal morphine occurs through opioid receptors in the spinal cord, the effect of rostral spread is attributed to the emergence of undesirable effects. However, systemic absorption factor also disappears when using intrathecal administration. The mechanism to explain the impact of intrathecal administration on PDPH is unclear. In another randomized controlled trial, a single dose of 150 µg administered after accidental dural puncture with a 17 G needle shortly after delivery did not decrease the incidence or severity of PDPH. The etiology of PDPH is multifactorial. That study does not support the clinical usefulness of prophylactic intrathecal morphine after accidental dural puncture, as in our study.^12^

The administration of neuraxial morphine in postoperative analgesia is considered the gold standard, provides superior analgesia compared to systemic practice and does not increase the risk of respiratory depression. With the increased dose, the action time is prolonged, and the maternal side effects increase. However, the perioperative respiratory monitoring period and frequency should be determined based on postoperative complications according to the intrathecal or epidural morphine dose in at-risk patients in particular (cardiopulmonary/neurological comorbidity, obesity (BMI >40), obstructive sleep apnea, sedative agent or chronic opioid use, and magnesium use in preeclamptic pregnancy). Low-dose morphine administration can provide effective analgesia and minimizes undesirable side effects such as itching, nausea-vomiting, and respiratory depression when combined with multimodal analgesics (non-steroidal anti-inflammatory drugs, acetaminophen, etc.). The recommended intrathecal low dose of morphine in healthy people is >0.05 to ≤0.15 mg, and the epidural dose is <1 mg to >3 mg.^2^

In our clinic, we used fentanyl and a combination of fentanyl-morphine as adjuvant. The sedation scales were similar in the postoperative follow-up, and no patients experienced respiratory depression. The analgesia duration was prolonged with the increased dose, but with low doses of morphine, statistically significant results were achieved at the end of VAS in the 6^th^ and 24^th^ hours. Cesarean section is the most common surgery performed in women worldwide. Postoperative analgesia during cesarean section is important for the mother to be active to meet the needs of the newborn baby in the early period and to support mother-baby communication psychologically.^12,17^ The addition of intrathecal bupivacaine and opioids for spinal anaesthesia improves the intraoperative conditions and contributes to postoperative analgesia. The combination of intrathecal morphine and fentanyl is effective and safe in cesarean analgesia.^18^ Because of the lipophilic properties of fentanyl, it's effect begins early, and it is 10-20 times more effective than intravenous administration.^19^ Intrathecal morphine with an onset time of approximately 30 minutes contributes to postoperative analgesia. Adding lipophilic fentanyl to accelerate the slow action onset improves intraoperative analgesia, reduces the local anaesthetic need, and decreases intraoperative hypotension.^20,21^

In a meta-analysis by Sultan et al.^18^, analgesia duration and side effects of morphine at different doses [0.05-0.1 (low dose) and >0.1-0.25 mg morphine (high dose)] in cesarean section cases were analyzed. First analgesic requirement was longer in the high-dose morphine group. First analgesic requirement in the high-dose morphine group was between 13.8 hours and 39.5 hours, while it was between 9.7 hours and 26.6 hours in the low-dose group. Pain scores were not different between the different morphine consumption level groups in the 12^th^ hour and 24^th^ hour. Side effects, such as nausea, vomiting, and itching, were less common in the low-dose group. Having an Apgar score of <7 in the 1^st^ minute was not different between the groups.^18^

In the present study, intrathecal 0.1 mg morphine was administered, and the initial need for analgesia was between 1.5 hours and 7 hours in Group F and 2 hours to 11 hours in Group M. However, the first analgesic requirement was not statistically significant among the groups. Low pain score in Group M in the 6^th^ and 24^th^ hours was statistically significant. The need for postoperative meperidine was significantly lower in Group M. The APGAR score in the 1^st^ min was 8.51 ± 1.28 in Group F and 8.62 ± 0.945 in Group M, and there was no statistically significant difference between the groups. The patients with an APGAR score <7 in the 1^st^ min were included in Group F (n = 3) and Group M (n = 1), and those with pH <7.2 were included in Group F (n = 1) and Group M (n = 1). While low doses of morphine contributed to the reduction of postoperative analgesia and meperidine consumption, no adverse effects were observed in the newborns.

### Study Limitations

Our study limitations were that this study was retrospective, and it was a single-centered study. Despite being retrospective, cesarean section patients are closely monitored for postspinal headaches and complications in our clinic, as obstetric patients are at risk for PDPH. Five-day follow-up forms with a high frequency of routine first 24-hour follow-ups were created for the patients. VAS scores were based on the subjective evaluations of the patients, and the evaluations on the 5^th^ day were completed by telephone calls. Our original estimated sample size could not be reached due to the retrospective nature of the study.

## Conclusion

In conclusion, there was no protective efficacy of low-dose morphine in reducing PDPH incidence and pain severity. Low-dose intrathecal morphine is an efficient and effective component of multimodal analgesia that does not increase maternal and fetal side effects in cesarean section patients. Further studies are needed to evaluate the effect of morphine on PDPH at different doses and with different routes of neuroaxial administration.

## Figures and Tables

**Table 1:**
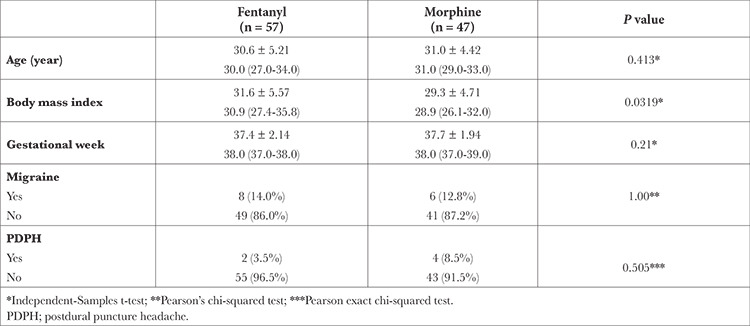
Demographic Data

**Table 2:**
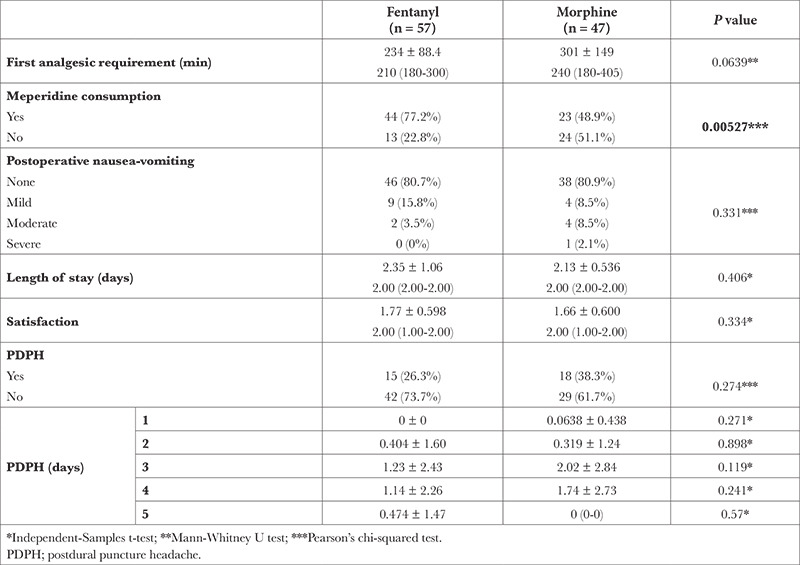
Need for Postoperative Analgesia
